# Development of Formulas for Calculating L3 Skeletal Muscle Mass Index and Visceral Fat Area Based on Anthropometric Parameters

**DOI:** 10.3389/fnut.2022.910771

**Published:** 2022-06-17

**Authors:** Wei Ji, XiangLiang Liu, Yiqun Zhang, Yixin Zhao, YuWei He, JiuWei Cui, Wei Li

**Affiliations:** Center of Cancer, The First Affiliated Hospital of Jilin University, Changchun, China

**Keywords:** cancer, nutrition, skeletal muscle mass, visceral fat area, formula

## Abstract

**Background:**

The anthropometric index is not accurate but shows a great advantage in accessibility. Simple body composition formulas should be investigated before proceeding with the universal nutrition screening.

**Materials and Methods:**

Clinical data of patients with a malignant tumor of the digestive system were collected. SliceOmatic 5.0 software (TOMOVISION, Canada) was used to analyze abdominal CT images and taken as references. A linear regression analysis was adopted to establish the formula for calculating skeletal muscle index (SMI) and visceral fat area (VFA). In addition, the relweights function was adopted to measure the contribution of each variable.

**Results:**

In total, 344 patients were divided into the training set and 134 patients into the validation set. The selected formulas were SMI.pre = 0.540 × weight (kg) – 0.559 × height (cm) – 13.877 × sex (male = 1, female = 2) + 123.583, and VFA.pre = 5.146 × weight (kg) – 2.666 × height (cm) + 1.436 × age (year) + 134.096, of which the adjusted *R*^2^ were 0.597 and 0.581, respectively. The “weight” explained more than 80% of *R*^2^ in the prediction of VFA. In addition, “sex” occupied approximately 40% of *R*^2^ in the prediction of SMI. The paired *t*-test showed no significant difference between the real measured indices and the predicting ones (*p* = 0.123 for SMI and *p* = 0.299 for VFA). The logistic regression analysis exhibited similar diagnostic efficacy of the real measured parameters and formulas.

**Conclusion:**

The SMI and VFA formulas were developed through basic indices, such as weight, height, sex, and age. According to the contribution of each variable, weight should always be focused on preserving appropriate muscle and adipose tissue.

## Introduction

Nutritional status is defined as “the condition of the body, resulting from the balance of intake, absorption, and utilization of nutrients and the influence of particular physiological and pathological status,” which is the foundation of all activities (1). Malnutrition, in all its forms, includes undernutrition (wasting, stunting, and underweight), inadequate vitamins or minerals, overweight or obesity, and diet-related non-communicable diseases. Malnutrition results in disease and often includes cardiovascular diseases, certain types of cancer, and diabetes. Among these, cancer has been a leading cause of death worldwide, accounting for approximately one in six deaths. According to statistics from the World Health Organization (WHO), approximately one-third of deaths from cancer are due to tobacco use, high body mass index (BMI), alcohol consumption, low fruit and vegetable intake, and lack of physical activity, which are closely linked to malnutrition. Besides, undernutrition is common in cancer. The Investigation on Nutrition Status and its Clinical Outcome of Common Cancers (INSCOC) project reported that 40–80% of patients with cancer are diagnosed with undernutrition, and 20% of patients with cancer die due to undernutrition (2). Thus, the assessment of nutritional status should be universal and paid attention, which has major relevance to clinical practice for individual and public health (3).

There have been various tools for nutrition assessment. Body composition, which precisely distinguishes body components into categories, such as muscle tissue, adipose tissue, and bone, is demonstrated to be instructive in clinical nutrition assessment, drug dosage, adverse events management, and prognosis prediction. Anthropology is the most traditional way with indices, such as height, weight, waist circumference (WC), and BMI. A single anthropometric index is not accurate but shows a great advantage in accessibility. Thus, iconography is proposed and regarded as the most accurate and reliable technique by most guidelines, such as the Asian Working Group of Sarcopenia (AWGS) (4), the European Working Group on Sarcopenia in Older People (EWGSOP) (5), and the Foundation for the National Institutes of Health (FNIH) Sarcopenia Project (6). Muscle mass is a part of body compositions, and the loss of muscle mass indicates a worse quality of life and deteriorated clinical outcomes. Appendicular skeletal muscle mass (ASM) is the classical parameter of skeletal muscle mass and has been recommended by multiple guidelines. Martin et al. (7) proposed skeletal muscle index (SMI) at the third lumbar vertebra as a nutritional indicator, which behaved well in the detection of occult muscle depletion compared with traditional nutritional indicators, such as BMI and weight loss. Then SMI and visceral fat area (VFA) at the third lumbar vertebra have been demonstrated as substitutes for whole-body composition and have been associated with clinical outcomes, which were gradually adopted (8, 9). Another reason for the extensive use of the third lumbar vertebra is its inclusion in typical abdominal CTs. Zhuang et al. (10) reported that SMI is an independent predictor of severe postoperative complications [odds ratio (*OR*) = 3.010, *p* < 0.001] and long-term survival [hazard ratio (*HR*) = 1.653, *p* < 0.001] after radical gastrectomy for gastric cancer. Li et al. proposed cutoffs of VFA in sarcopenic obesity and demonstrated its unfavorable impact on survival (*HR* = 2.772, *p* < 0.001) (9). However, equipment and technical requirements are obvious drawbacks to iconography, which makes it seem unreasonable under the circumstance of universal and quick nutrition assessment. The updated 2019 AWGS consensus cited the formula of calculating AMS simply by anthropometric indices, that is, ASM (kg) = 0.193 × weight (kg) + 0.107 × height (cm) – 4.157 × gender (male = 1, female = 2) – 0.037 × age (year) – 2.631, which was also demonstrated to be effective in the assessment of body composition (4). Sarcopenia diagnosed accordingly was an independent factor of 3-year mortality [*HR* = 2.49, 95% confidential interval (*CI*): 1.25–4.95] and readmission (*HR* = 1.81, 95% *CI* 1.17–2.80) in a population of elderly inpatients in acute care wards (11).

To shed light on this context, more simple body composition formulas should be explored to proceed with the universal nutrition screening, especially in rural districts where iconographic equipment or technology are unavailable. Herein, taking CT measured SMI and VFA as a reference, formulas of SMI and VFA by the simple anthropometric index were first established and verified in 478 patients with malignant tumors of the digestive system. In addition, the contribution of each variable involved was determined, which explicitly reminds health providers of the focus on multivariables.

## Patients and Methods

The study protocol adhered to the Declaration of Helsinki and was approved by the Ethics Committee of the First Hospital of Jilin University (2017-362).

### Patients

The clinical data of patients with a malignant tumor of the digestive system who were admitted into the First Affiliated Hospital of Jilin University from November 2011 to December 2018 were collected. The inclusion criteria were as follows: (1) adults > 18 years old and (2) abdominal CT scans to be obtained. The exclusion criteria involved patients with incomplete data.

Clinical data were collected by trained personnel for each participant. (1) General characteristics include age, sex, smoking history, alcohol drinking, and comorbidities (diabetes and hypertension). (2) Anthropometric measurements include BMI: patients needed to empty their bladder and fast for 2 h before the measurement. During the measurement, patients took off their shoes and wore light clothing. Height and weight were measured accurately to 1 cm and 0.1 kg. BMI (kg/m^2^) was calculated by weight (kg)/height (m)^2^. For mid-arm circumference (MAC) and triceps skinfold thickness (TSF), MAC of the non-dominant side was measured to the nearest 0.5 cm using a non-elastic tape when patients were standing, and TSF at the same place was measured accurately to 1 mm using vernier calipers. For hand-grip strength (HGS), patients were seated with 90 degree elbow flexion and the grip strength of the dominant hand was measured to the nearest 1 kg at least two times with the Jamar dynamometer, and the maximum reading was recorded. For waist circumference (WC), the non-elastic tape was placed at the umbilicus and was encircled at the abdomen parallel to the ground, closing to the skin without squeezing the skin. WC was measured accurately to 0.5 cm. For maximum calf circumference (CC), the maximum circumference of the left calf was measured with a non-elastic tape when standing, which was accurate to 0.5 cm. (3) Serum albumin concentration, serum C-reaction protein (CRP), and triglycerides (TGs) were assessed through laboratory examinations. (4) The patient-generated subjective global assessment (PG-SGA) was used as the evaluation scale.

### Computer Tomography Indices

**FIGURE 1:**
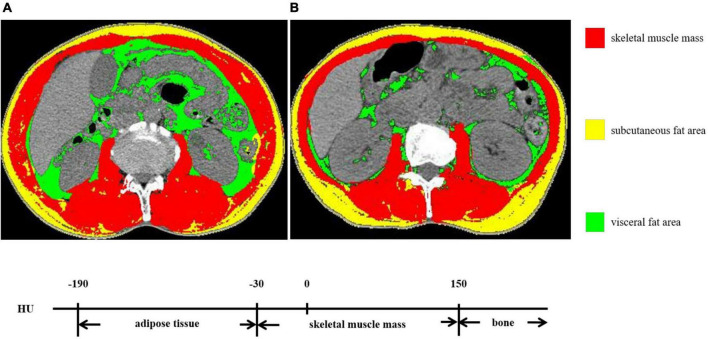
The skeletal muscle mass and visceral fat area (VFA) at the third lumbar vertebra. **(A,B)** exhibited two patients with the same BMI but different body compositions.

SliceOmatic 5.0 software (TOMOVISION, Canada) was used to analyze abdominal CT images. According to the voxel values, –29 to + 150 Hu was identified as skeletal muscle mass and −190 to −30 Hu was identified as adipose tissue mass. Then, the skeletal muscle area (SMA) and VFA at the third lumbar vertebra were sketched. The SMA includes the psoas major, the erector spinae, the quadratus lumborum, the transverse abdominis, the external oblique, and the internal oblique. The VFA represents the intra-abdominal adipose tissue. Figure 1 displays the diagrammatic sketch illustrating two patients with the same BMI but different body compositions. SMI (cm^2^/m^2^) = SMA/height^2^ (m^2^).

### Statistical Analysis

Data were analyzed by SPSS for Windows version 26.0 (IBM SPSS Statistics, IBM Corp., Armonk, NY) and R version 4.0 (R Foundation for Statistical Computing, Vienna, Austria).

The training set and validation set were separated randomly at a ratio of 7:3 (12). The Kolmogorov–Smirnov test was used to confirm normal distributions of continuous data. An independent *t*-test was used for normally distributed data. Counting data were examined by using the chi-square test. Pearson’s correlation analysis was adopted. Multi-collinearity was tested by linear regression analysis, and the variance inflation factor (VIF)>10 was considered as the existence of collinearity. The linear regression analysis was adopted to establish the formula for calculating SMI and VFA. Then, the paired *t*-test was used to examine the accuracy of the regression formula in the training set and validation set. The logistic regression analysis was adopted to examine the efficacy of the formulas in malnutrition (PG-SGA ≥ 9). The *p* < 0.05 was taken to indicate statistical significance.

## Results

### Basic Characteristics of Involved Patients

In total, 478 patients were involved in the study. Out of which, 344 patients were separated into the training set and 134 patients into the validation set. All basic characteristics were consistent between the two sets (*p*>0.05) (Table 1). The SMI was 45.40 cm^2^/m^2^ ± 9.17 cm^2^/m^2^ in the training set and 44.70 cm^2^/m^2^ ± 9.58 cm^2^/m^2^ in the validation set (*p* = 0.451). The VFA was 101.13 cm^2^ ± 66.28 cm^2^ in the training set and 102.41 cm^2^ ± 69.15 cm^2^ in the validation set (*p* = 0.848). The Pearson correlation coefficient was 0.725 between ASMI and SMI (*p* < 0.001).

**TABLE 1:** Basic characteristics of involved participants.

Characteristics	Training set	Validation set	t/χ^2^	*p*
Age(year)	58.76 ± 10.53	59.46 ± 9.53	–0.680	0.497
Sex			0.755	0.385
Male	218(45.6)	88(18.4)		
Female	116(24.3)	56(11.7)		
Height(cm)	166.42 ± 8.37	165.42 ± 8.36	1.456	0.146
Weight(kg)	63.02 ± 11.76	62.06 ± 11.65	0.825	0.410
BMI(kg/m^2^)	22.67 ± 3.45	22.62 ± 3.23	0.147	0.883
Smoking			2.416	0.120
Yes	165(34.5)	60(12.6)		
No	169(35.4)	84(17.6)		
Drinking			0.003	0.958
Yes	92(19.2)	40(8.4)		
No	242(50.6)	104(21.8)		
Comorbidities			1.817	0.969
Diabetes	18(3.8)	10(2.1)		
Hypertension	47(9.8)	20(4.2)		
MAC(cm)	26.36 ± 3.38	26.17 ± 3.09	0.582	0.561
TSF(mm)	16.24 ± 6.38	17.25 ± 10.73	–1.276	0.203
HGS(kg)	26.29 ± 10.62	24.86 ± 10.62	1.393	0.164
WC(cm)	82.31 ± 10.01	81.94 ± 10.09	0.302	0.763
CC(cm)	33.62 ± 4.28	33.15 ± 3.75	1.138	0.256
Albumin(g/L)	37.19 ± 5.34	37.81 ± 4.91	–1.187	0.236
CRP(mg/L)	20.51 ± 32.74	21.36 ± 41.34	–0.191	0.849
TG(moml/L)	1.37 ± 0.86	1.49 ± 1.05	–0.939	0.348
ASMI(kg/m^2^)	6.96 ± 1.06	6.86 ± 1.14	0.845	0.398
SMI(cm^2^/m^2^)	45.40 ± 9.17	44.70 ± 9.58	0.754	0.451
VFA (cm^2^)	101.13 ± 66.28	102.41 ± 69.15	–0.191	0.848

*BMI: body mass index; MAC: mid-arm circumference; TSF: triceps skinfold thickness; HGS: hand grip strength; WC: waist circumference; CC: maximum calf circumference; CRP: C-reaction protein; TG: triglycerides; ASMI: appendicular skeletal muscle mass index; VFA: visceral fat area; SMI: skeletal mass index at the third lumbar vertebra; CT: computer tomography.*

### Linear Regression Model of Skeletal Mass Index and Visceral Fat Area

**FIGURE 2:**
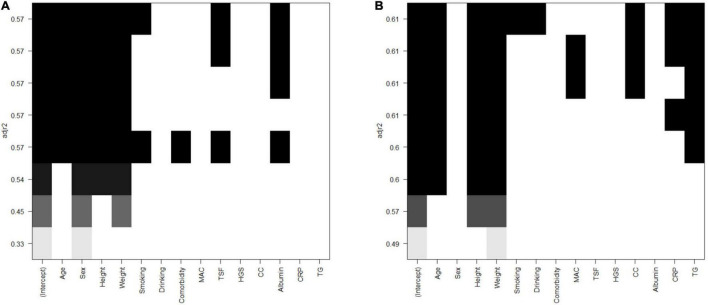
All subsets regression of SMI and VFA. **(A)** SMI. **(B)** VFA. SMI: skeletal mass index at the third lumbar; VFA: visceral fat area.

**FIGURE 3:**
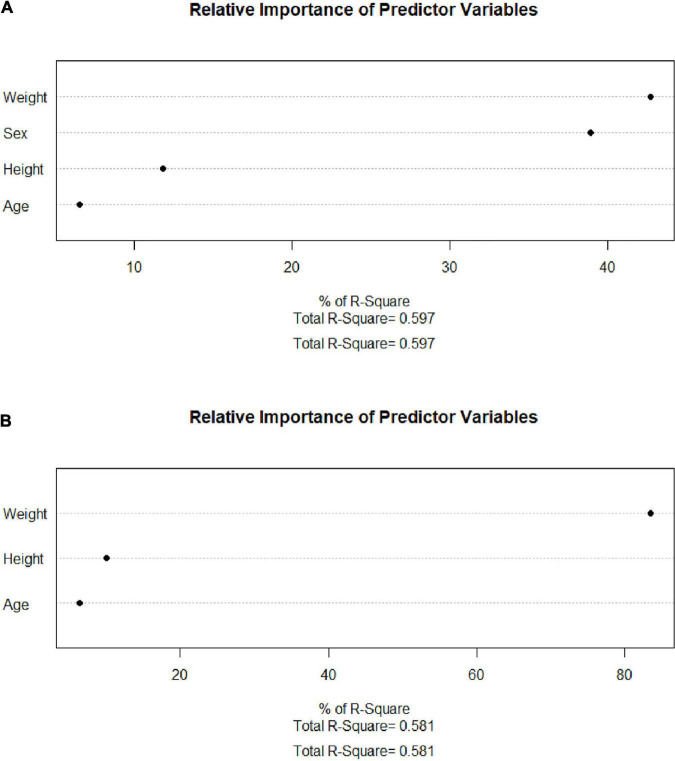
Relweights of each variable. **(A)** SMI. **(B)** VFA. SMI: skeletal mass index at the third lumbar; VFA: visceral fat area.

After collinearity diagnosis, BMI was excluded (VIF = 139.091). Then, all subset regression was committed and the results are displayed in Figure 2. The most appropriate model was primarily selected according to the adjusted *R*^2^ (which means how well the predictor variable explains the response variable) and the accessibility of the involved variables. Thus, the selected formula was SMI.pre = 0.540 × weight (kg) − 0.559 × height (cm) – 13.877 × sex (male = 1, female = 2) + 123.583 and VFA.pre = 5.146 × weight (kg) − 2.666 × height (cm) + 1.436 × age (year) + 134.096, of which the adjusted *R*^2^ were 0.597 and 0.581, respectively. Then, the relweights function (13) was adopted to measure the importance of each variable. As shown in Figure 3, the most important variable was “weight,” both in the prediction of SMI and VFA. Especially, the “weight” explained more than 80% of *R*^2^ in the prediction of VFA. In addition, “sex” should be paid attention to in the prediction of SMI, which occupied approximately 40% of R^2^. “Height” and “age” only occupied approximately 10% both in the formula of SMI and VFA.

### Validation of Predicting Models of Skeletal Mass Index and Visceral Fat Area

Paired *t*-test indicated that there was no significant difference between the real measured SMI and SMI.pre (SMI calculated by the formula proposed above) in the training set (*p* = 0.165), and the paired sample correlation was 0.726 (*p*<0.001). Additionally, the real measured VFA and VFA.pre (VFA calculated by the formula proposed above) were consistent (*p* = 0.388) with the paired sample correlation of 0.770 in the training set. In the validation set, the paired sample correlation was 0.789 (*p*<0.001) for SMI and 0.745 (*p*<0.001) for VFA, and no significant difference was observed between the real measured indices and the predicting ones (*p* = 0.123 for SMI and *p* = 0.299 for VFA) (Table 2).

**TABLE 2:** Validation of predicting models of SMI and VFA.

	Pearson correlation		Paired *t*-test	
	r	P	t	P
**Training set**				
SMI vs. SMI.pre*	0.726	<0.001	–1.391	0.165
VFA vs. VFA.pre^#^	0.770	<0.001	0.865	0.388
**Validation set**				
SMI vs. SMI.pre*	0.789	<0.001	–1.552	0.123
VFA vs. VFA.pre^#^	0.745	<0.001	1.042	0.299

*SMI.pre = 0.540* weight (kg)-0.559* height (cm) –13.877* sex (male = 1, female = 2) + 123.583.

^#^VFA.pre = 5.146* weight (kg)-2.666* height (cm) + 1.436* age (year) + 134.096. SMI: skeletal mass index at the third lumbar vertebra; VFA: visceral fat area.

### The Diagnostic Efficacy of the Established Models of Skeletal Mass Index and Visceral Fat Area

The thresholds of SMI and VFA were derived from previous studies. The cutoffs of SMI were 34.9cm^2^/m^2^ for women and 40.8cm^2^/m^2^ for men in sarcopenia (10), and the diagnostic consistency was 87.3%. The cutoffs of VFA were 61.2 cm^2^ for women and 75.2 cm^2^ for men (9) and the diagnostic consistency was 86.5%. The clinical events diagnosed by the real measured and predicted parameters are displayed in Table 3. No significant difference was detected between groups (*p*>0.05). Then, the logistic regression analysis was adopted to examine the efficacy of the real parameters and established models for detecting severe undernutrition (PG-SGA ≥ 9). The *OR*s were 2.327 (95% *CI* 1.986–2.873, *p*<0.001) for SMI and 2.106 (95% *CI* 1.735–2.494, *p*<0.001) for SMI.pre. When combined with low SMI and high VFA, which is referred to as sarcopenic obesity, the *OR*s were 3.172 (95% *CI* 2.416–3.928, *p*<0.001) for the real measured parameters and 2.743 (95% *CI* 2.016–3.748, *p*<0.001) for the predicted parameters.

**TABLE 3:** The clinical events diagnosed by real measured and predicted parameters and their nutritional status.

Characteristics	PG-SGA		χ^2^	P
	<9	≥ 9		
Sarcopenia			0.072	0.789
SMI (*n* = 87)	56(64.4)	31(35.6)		
SMI.pre* (*n* = 89)	59(66.3)	30(33.7)		
SOB^#^			0.035	0.851
Real (*n* = 13)	9(69.2)	4(30.8)		
Predicted (*n* = 11)	8(72.7)	3(27.3)		

*SOB: sarcopenic obesity. SMI: skeletal mass index at the third lumbar; PG-SGA: patient generated subjective global assessment. VFA: visceral fat area;*

*SMI.pre = 0.540* weight (kg)–0.559* height (cm) –13.877* sex (male = 1, female = 2) + 123.583.

^#^SOB was diagnosed by low SMI combined with high VFA. And VFA.pre = 5.146* weight (kg)-2.666* height (cm) + 1.436* age (year) + 134.096.

## Discussion

Undernutrition in patients with cancer is pretty common. The general prevalence of undernutrition is 40–80% in Chinese patients with cancer. Undernutrition could result from tumor-derived cytokine release, tumor mass effects, and side effects of cancer treatment. As a multifaceted disease, the symptoms of undernutrition vary from loss of appetite to sarcopenia and even cachexia. Undoubtedly, undernutrition significantly deteriorates function outcomes, survival, and increased additional costs (14). To deal with this, nutrition interventions are introduced. The prospective clinical trial EFFORT demonstrated that individualized nutritional support reduced the risk of mortality and improved functional and quality of life outcomes in cancer patients with increased nutritional risk (15). These data support universal malnutrition screening upon hospital admission followed by an individualized nutritional support strategy in these vulnerable patients to prevent adverse clinical outcomes associated with malnutrition, which was also recommended by the European Society for Clinical Nutrition and Metabolism (ESPEN) (16).

However, nutrition screening is far from ideal. Although the importance is already obvious, the practice of nutrition screening and malnutrition diagnosis is lacking (17). Li et al. (18) reported that only 0.5% of patients with cancer received nutrition screening at admission, but the prevalence of malnutrition was up to 44.9% actually in the same cohort. The dilemma resulted from multiple reasons. Nutritional scales are commonly used like malnutrition universal screening tool (MUST), nutrition risk score-2002 (NRS-2002), and the patient-generated subjective global assessment (PG-SGA) (19). However, the implementation needs trained personnel, compliance, and time costs. Body composition directly and individually reflects nutritional status. However, body composition analysis is complex and requires investment in personnel, equipment, and technology. Therefore, simplifying the method of body composition analysis will promote the accessibility of nutritional screening.

Herein, formulas calculating SMI and VFA all by anthropometric indices were established, examined, and validated. As displayed in Figure 2, the SMI model with the least variables was selected with the same predictive ability. Among the VFA models, the predictive ability is slightly sacrificed given the accessibility of variables. The VFA model with the largest *R*^2^ was not selected because the involvement of TG only increased about 1% of predictive ability. Thus, the finally selected formulas are SMI.pre = 0.540* weight (kg) − 0.559 × height (cm) – 13.877 × sex (male = 1, female = 2) + 123.583 and VFA.pre = 5.146 × weight (kg) − 2.666* height (cm) + 1.436* age (year) + 134.096. Furthermore, the relweights function reported novel information on these variables. Weight is the most important parameter, which occupies the largest share in VFA and SMI formula. Especially in the VFA formula, the relweight of weight is up to 80%. In fact, weight is considered a rough parameter of nutritional status and overall adiposity. Studies have suggested a beneficial effect of obesity, named the “obesity paradox.” The phenomenon was then clarified that it was the increased muscle that dominated the benefits, whereas the accumulated visceral fat defeated the benefits (20). In addition, it has been demonstrated that, compared with normal-weight individuals, subjects with obesity have greater thigh muscle volume, increased cross-sectional area of type I skeletal muscle fibers, increased muscle lipid content, and a lower muscle quality (21–23). These findings remind the importance of weight management. Appropriate weight means suitable muscle mass, muscle quality, and adipose tissue. In addition, sex should be emphasized in the assessment of muscle mass for the relative weight of approximately 40% in SMI. It should be noted that this result does not indicate that women are more prone to muscle depletion because of different cutoff values. Zhuang et al. (10) reported the appropriate threshold of SMI in sarcopenia, which was 34.9 cm^2^/m^2^ for women and 40.8 cm^2^/m^2^ for men. It was reported that men were more vulnerable to sarcopenia than women. Bianchi L et al. (24) reported that the prevalence of sarcopenia was 36.5% in men and 32.9% in women. Since gender is also an included variable, the gender differences should be paid attention to at least in epidemiological investigations and public health management.

Based on the principle of providing a universal and practicable method, only simple and easily available parameters were involved. From the perspective of convenient and universal nutrition screening, the formulas established should be a step forward with good consistency and no statistically significant difference between the calculated values and real values. Since the formulas were established and validated, we also recommended the same thresholds for the real measured and predicted SMI and VFA in clinical settings. However, the formulas were not proposed to substitute the ever-changing body composition tools. There are limitations. Although the training cohort and validation cohort were set to guarantee the accuracy of the formula, the formulas must be validated through large cohorts from multi-centers. As a subject still under exploration with limited sample size, it is difficult for the involved population to represent the whole. Herein, patients with digestive system cancer were applicable. The results are not necessarily generalizable to other populations. Verifying the formulas in healthy populations and populations with various diseases would further enhance the credibility.

In conclusion, SMI and VFA, the two important parameters in body composition, were calculated by basic indices, such as weight, height, sex, and age with good consistency. It appears to be a simple and valid tool for assessing the body composition of patients with cancer. The contribution of each variable involved was also reported, which explicitly reminds health providers to take care of important indices, such as weight. This project was potentially able to promote the implementation of nutrition screening.

## Data Availability Statement

The original contributions presented in this study are included in the article/supplementary material, further inquiries can be directed to the corresponding authors.

## Ethics Statement

The studies involving human participants were reviewed and approved by the Ethics Committee of the First Hospital of Jilin University. Written informed consent for participation was not required for this study in accordance with the national legislation and the institutional requirements.

## Author Contributions

WJ and XL designed the study. YQZ and YXZ collected and analyzed the data. YH analyzed the data. JC and WL critically revised the manuscript. All authors read and approved the final manuscript.

## Conflict of Interest

The authors declare that the research was conducted in the absence of any commercial or financial relationships that could be construed as a potential conflict of interest.

## Publisher’s Note

All claims expressed in this article are solely those of the authors and do not necessarily represent those of their affiliated organizations, or those of the publisher, the editors and the reviewers. Any product that may be evaluated in this article, or claim that may be made by its manufacturer, is not guaranteed or endorsed by the publisher.
